# Efficacy and safety of different doses of cytarabine in consolidation therapy for adult acute myeloid leukemia patients: a network meta-analysis

**DOI:** 10.1038/s41598-017-10368-0

**Published:** 2017-08-25

**Authors:** Di Wu, Chongyang Duan, Liyong Chen, Size Chen

**Affiliations:** 1 Guangdong Province Key Laboratory for Medical Molecular Diagnostics, China-America Cancer Research Institute, Dongguan Scientific Research Center, Guangdong Medical University, Dongguan, 523808 China; 2 Department of Biostatistics, Southern Medical University, Guangzhou, 510515 China; 3 Central Laboratory, the First Affiliated Hospital of Guangdong Pharmaceutics University, Guangzhou, 510405 China

## Abstract

Cytarabine (Ara-C) in consolidation therapy played important role in preventing relapses for AML patients achieved complete remission, but the optimum dose remains elusive. In this network meta-analysis, we compared benefit and safety of high-, intermediate- and low-dose Ara-C [HDAraC (>2 g/m^2^, ≤3 g/m^2^ twice daily), IDAraC (≥1 g/m^2^, ≤2 g/m^2^ twice daily) and LDAraC (<1 g/m^2^ twice day)] in consolidation, based on ten randomized phase III/IV trials from 1994 to 2016, which included 4008 adult AML patients. According to the results, HDAraC in a dosage of 3 g/m^2^ twice daily significantly improved disease-free survival (DFS) compared with IDAraC [hazard rate (HR) 0.87, 95% CrI 0.79–0.97) and LDAraC (HR 0.86, 95% CrI 0.78–0.95). Subgroup analysis further showed that the DFS advantage of HDAraC is focused on the patients with favorable cytogenetics, but not the other cytogenetics. Compared with LDAraC, HDAraC (HR 6.04, 95% CrI 1.67–21.49) and IDAraC (HR 3.80, 95% CrI 1.05–12.85) were associated with higher risk of grade 3–4 non-haematological toxicity. However, no significant difference between HDAraC and IDAraC was found. These findings suggest that Ara-C in a dosage of 3 g/m^2^ twice daily provides maximal anti-relapse effect.

## Introduction

With an incidence of 3–4 every 100,000 people, acute myeloid leukemia (AML) is the most common acute leukemia in the world. Standard induction chemotherapy based on the cytarabine (Ara-C) and anthracyclines helps 60–80% younger adult patients to achieve complete remission (CR)^1–3^. However, only one-third of these patients remain disease-free for over 5 years and all the CR ones will eventually relapse without further therapy^4, 5^. Appropriate postremission therapy is thus essential.

Except for stem-cell transplantation, Ara-C-based consolidation chemotherapy were proven mandatory in preventing relapses after achieving a first CR^6^. As Cancer and Leukemia Study Group B (CALGB) 8525 trial revealed that high-dose cytarabine (HDAraC: 3 g/m^2^ twice daily over 3 days) regimen was superior to two low-dose cytarabine regimens (LDAraC: 0.4 g/m^2^ and 0.1 g/m^2^ over 5 days) for untreated AML patients, repetitive cycles of single-agent HDAraC was widely adopted as a standard post-remission chemotherapy^7^. After that, four multicenter, randomized controlled trials (RCTs) respectively reported that investigational multiagent consolidation chemotherapies involved LDAraC (0.1–1 g/m^2^ over 3–5 days) failed to show an improvement in any survival endpoints when compared with HDAraC^8–11^. And in the Study Alliance Leukemia (SAL) AML 96 and AML 2003 trials, which performed to compare the intermediate-dose cytarabine (IDAraC: 1 g/m^2^ over 6 days and 1.5 g/m^2^ over 3 days) with HDAraC again failed to find any survival advantages^10, 12^. Even though Ara-C is the most active compound in consolidation chemotherapy, but the question of optimum dose of Ara-C remains unanswered^3, 13^.

In order to address this issue prospectively, we conducted a network meta-analysis including ten randomized phase III/IV trials in which Ara-C was given with different doses in consolidation.

## Results

### Characteristics of included trials and patients

**Figure 1:**
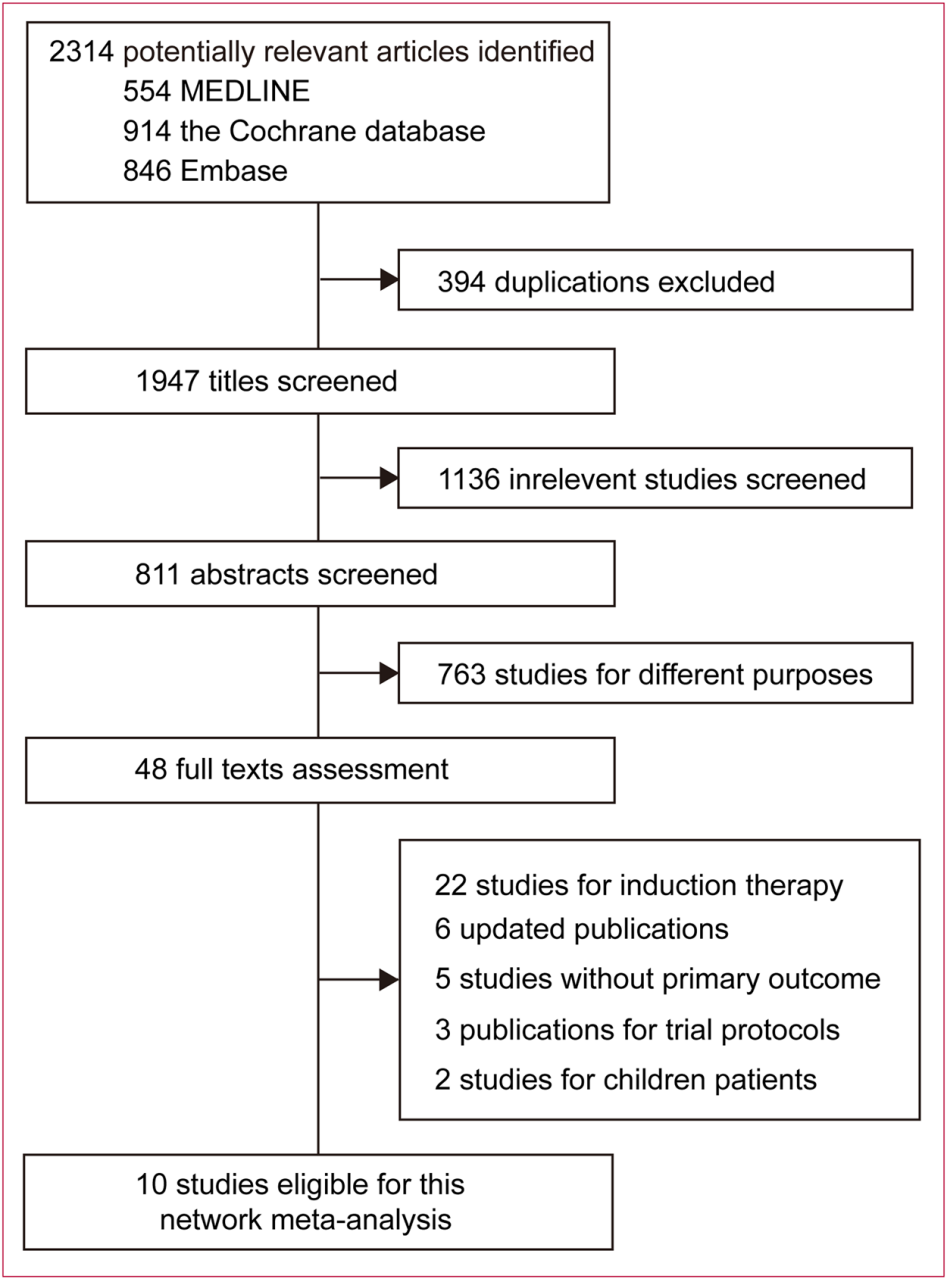
Identification of eligible randomized trials.

We identified 2314 records for reviewing the titles and abstracts (Fig. 1). After initial exclusion, we retrieved the full texts for further assessment. Ten multicenter, open-labeled, randomized phase III/IV trials were finally included for this meta-analysis (Table 1), with a total of 4008 AML patients randomized to receive one of the three dose range of Ara-C in consolidation: high-dose Ara-C [HDAraC (>2 g/m^2^, ≤3 g/m^2^ twice daily)], intermediate-dose Ara-C [IDAraC (≥1 g/m^2^, ≤2 g/m^2^ twice daily)] and low-dose Ara-C [LDAraC (<1 g/m^2^ twice day)]. These trials included a mean of 411.6 (SD 254.6, range 131–781) primary and second AML patients (including patients with myelodysplastic syndromes) aged 15–85 years with adequate organ function.

**Table 1:** Summary of studies included in meta-analysis.

Study	Design	Period	Entry Criteria	Size	Age (years)	Induction Therapy	CR (%)	Single Ara-C dose		Cumulative Ara-C dose	Medium follow-up (Month)
HDAraC vs IDAraC											
MRC AML15	Openflabel, multicenter phase III	2002–2009	Primary or secondary AML including MDS; no pregnancy; aged 15–60 years	329/328	48 (15–69)	DA or ADE or FLAG-Ida × 1–2	78–82	(3 g/m^2^ every 12 h on days 1, 3, 5) × 2 vs (1.5 g/m^2^ every 12 h on days 1, 3, 5) × 2		36 g vs 18 g	67 (2.4–114)
SAL AML2003	Open-label, multicenter phase III	2003–2009	Primary or secondary AML, or refractory anemia with excess blasts (RAEB2); aged 16–60 years	251/254	47 (18–60)	DA × 2	65	(3 g/m^2^ every 12 h on days 1, 3, 5) × 3 vs (1 g/m^2^ every 12 h on days 1–5/6) × 2		54 g vs 20–22 g	NR
SAL AML96	Open-label, multicenter phase IV	1996–2003	Primary or secondary AML; aged 15–64 years	363/382	47 (15–60)	MAV-MAMAC × 1	66	(3 g/m^2^ every 12 h on days 1–6) × 1 vs (1 g/m^2^ every 12 h on days 1–6) × 1		36 g vs 12 g	99.6 (NR)
IDAraC vs LDAraC											
SWOG 8601	Open-label, multicenter phase III	1986–1993	Primary AML; no MDS; aged < 65 years;	78/53	45 (15–60)	DA	58	(2 g/m^2^ every 12 h on days 1–5) × 1 vs (0.2 g/m^2^/d on days 1–7) × 2		20 g vs 2.8 g	51 (NR)
JALSG AML201	Open-label, multicenter phase III	2001–2005	Primary AML with enough function of major organs; no MDS; aged 15–64 years	389/392	47 (15–64)	DA or AI × 1–2	78	(2 g/m^2^ every 12 h on days 1–5) × 3 vs (0.2 g/m^2^/d on days 1–5) × 4		60 g vs 4 g	48 (5–78)
EORTC & GIMEMA AML8B	Open-label, multicenter phase III	1986–1993	Primary AML; no APL; absence of irreversible major organ failure; aged 46–60	158/157	NR (46–60)	DA × 1–2	61	(0.5 g/m^2^ every 12 h on days 1–6) × 1 + (2 g/m^2^ every 12 h on days 1–4) × 1 vs (0.2 g/m^2^/d on days 1–7) × 1		22 g vs 1.4 g	225 (NR)
HDAraC vs LDAraC											
SAKK 1985	Open-label, multicenter phase III	1985–1992	Primary AML (FAB Ml-6); aged 15–65 years	70/67	45 (16–61)	DA × 1 + (Amsacrine + VP-16) × 1	61	(3 g/m^2^ every 12 h on days 1–6) × 1 vs (0.1 g/m^2^/d on days 1–7) × 1		36 g vs 0.7 g	72 (NR)
CALGB 8525	Open-label, multicenter phase III	1985–1990	Primary AML; no prior MDS, uncontrolled infection; aged 16–86 years	187/206/203	52 (16–86)	DA × 1–2	64	(3 g/m^2^ every 12 h on days 1, 3, 5) × 4 vs (0.4 g/m^2^/d on days 1–5) × 4		72 g vs 8 g vs 2 g	52 (NR)
ALFA 9802	Open-label, multicenter phase III	1999–2006	Primary AML; no APL; absence of irreversible major organ failure; aged 15–50 years	117/120	46 (17–50)	DA–MTZA × 1	89	(3 g/m^2^ every 12 h on days 1, 3, 5) × 4 vs (0.5 mg/m^2^/d on days 1–3) × 2		72 g vs 3 g	60 (NR)
ALLG M7	Open-label, multicenter phase III	1995–2000	Primary AML; absence of irreversible major organ failure; aged 15–60 years	99/103	41 (15–60)	High-dose Ara-C + IA + VP-16 × 1–2	80	(3 g/m^2^ every 12 h on days 1, 3, 5, 7) × 1 vs (0.1 g/m^2^/d on days 1–5) × 2		24 g vs 0.5 g	45 (NR)

MRC, Medical Research Council; SAL, Study Alliance Leukemia; EORTC, European Organization for Research and Treatment of Cancer; GIMEMA, Gruppo Italiano Malattie Ematologiche Maligne dell’Adulto; JALSG, Japan Adult Leukemia Study; Group CALGB, Cancer and Leukemia Group B; ALFA, Acute Leukemia French Association; ALLG, Australasian Leukaemia and Lymphoma Group; AML, acute myeloid leukemia; MDS, myelodysplastic syndromes; Ara-C, cytarabine; DA, daunorubicin and cytarabine; ADE, cytarabine, daunorubicin, and etoposide; FLAG-Ida, fludarabine, cytarabine, granulocyte colony-stimulating factor, and idarubicin; MAV–MAMAC, mitoxantrone, standard-dose cytarabine, etoposide – intermediate-dose cytarabine, amsacrine; AI: cytarabine, idarubicin; MTZA, mitoxantrone, cytarabine; IA, idarubicin; VP–16, etoposide; NR, not reported.

During induction therapies, DA (daunorubicin and Ara-C) or IA (idarubicin and Ara-C) based strategies with conventional Ara-C doses (at 0.1–0.4 g/m^2^)^14^ were performed in seven out of ten trials^7, 9–11, 15–17^. The other three trials that used low-to-intermediate-dose, intermediate-dose and high-dose Ara-C, but not conventional doses, were excluded in sensitivity analysis^8, 12, 18^. During consolidation therapies, CR patients were randomized to receive the three dose ranges of Ara-C, resulting in corresponding cumulative dose ranges as follows: 24 to 72 g for HDAraC, 12 to 22 g for IDAraC and 0.5 to 8 g for LDAraC. To be noted, in AML 201, a single dose of 2 g/m² twice daily was divided into LDAraC. However, relatively more frequent injections of Ara-C in this trial resulted in a cumulative dose of 60 g, which belonged to high cumulative dose range^9^. Therefore, AML 201 was divided into HDAraC-used trials in sensitivity analysis.

### Risk of bias in included studies

All included trials have been published as full manuscripts and most of them have a low risk of bias (Fig. S1). The sequence was adequately generated in nine out of ten trials^7–12, 16–18^ and was not report in one trial^15^; we judged the quality of this trial as unclear risk. Allocation was adequately concealed in six out of ten trials^7, 8, 11, 16–18^ and was not reported in four trials^9, 10, 12, 15^; we judged the quality of these trials as unclear risk. Given the unambiguous study treatments and “strict” endpoints (DFS and OS), we did not anticipate any impact of lack of blinding on outcomes. For treatment-related toxicity, all studies used pre-planned standard grading methods and uniform follow-up scheme for all study groups. We judged all the ten trials as low risk. In all the ten trials, intention to treat principle was followed and the drop-outs were less than 10%. All the pre-planned outcomes were addressed.

### Pairwise meta-analysis

**Figure 2:**
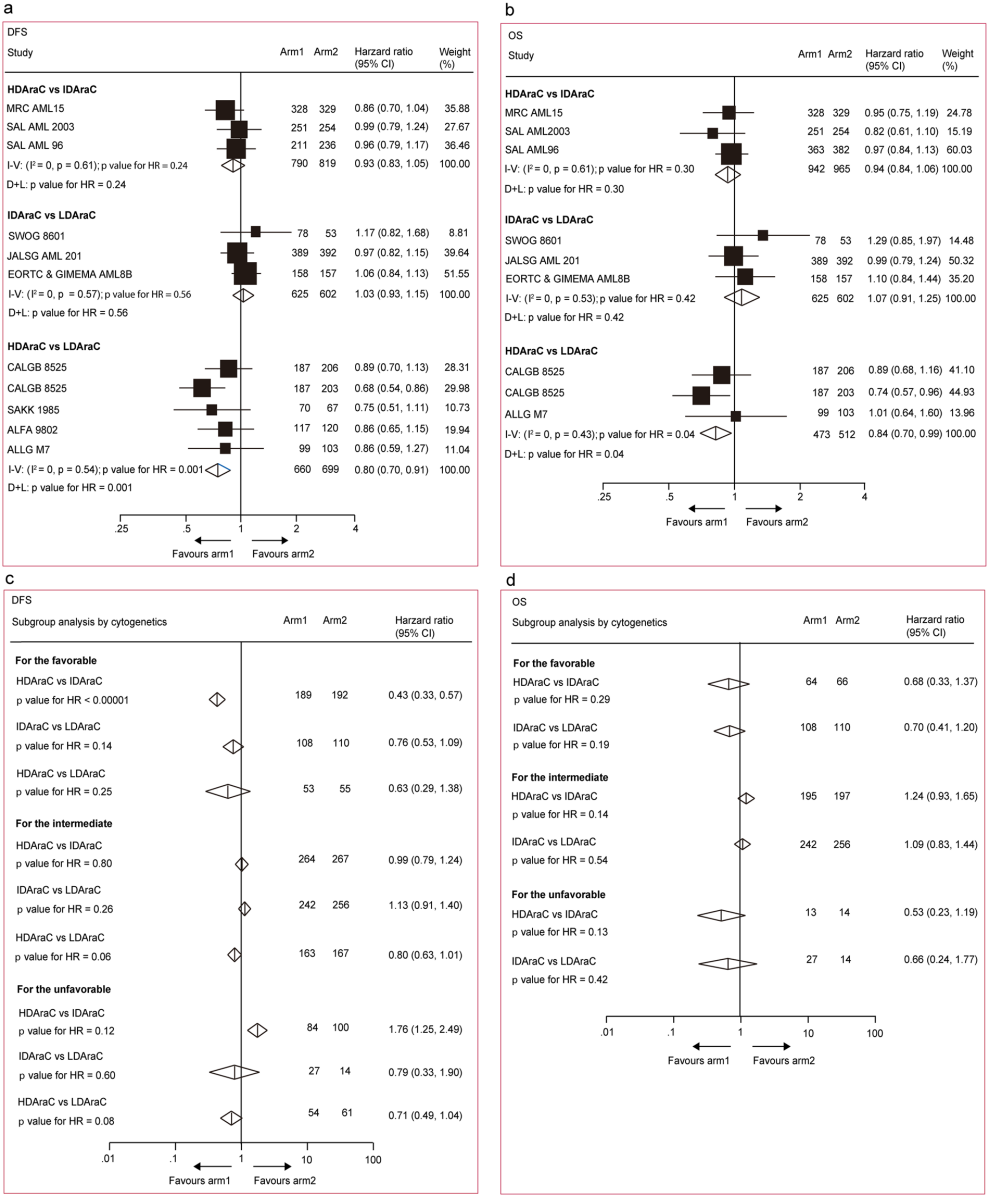
Direct meta-analysis for disease-free survival and overall survival. (**a**) and (**b**) All patients. (**c**) and (**d**) According to cytogenetic risk groups. The size of the boxes is proportional to the amount of data contained in each data line. The bars indicate 95% confidence intervals (CIs). HDAraC, high-dose cytarabine (>2 g/m^2^, ≤3 g/m^2^ twice daily); IDAraC, intermediate-dose cytarabine (≥1 g/m^2^, ≤2 g/m^2^ twice daily); LDAraC, low-dose cytarabine (<1 g/m^2^ twice daily); I–V = inverse variance. D + L = DerSimonan and Laird.

HRs for DFSs and OSs could be respectively estimated in all the ten trials including 4008 patients^7–12, 15–18^ and eight trials including 3932 patients^8–12, 15–17^. In pairwise comparisons across all cytogenetics (Fig. 2a and b), when compared with LDAraC, HDAraC in consolidation significantly improved DFS (HR 0.80, 95% CI 0.70–0.91, *p* = 0.001) and OS (HR 0.84, 95% CI 0.70–0.99, *p* = 0.04). For both endpoints, no significant difference was found in other comparisons. The same results were acquired when used both fixed and random effect models because no significant heterogeneity was found in all comparisons (*I* ^2^ = 0). In subgroup analysis stratified by cytogenetics (Fig. 2c and d), HRs for DFS was available in five studies including 2406 patients^7, 9, 10, 12, 16^. Compared with IDAraC, HDAraC in consolidation significantly benefited DFS (HR 0.43, 95% CI 0.33–0.57, p < 0.00001) for the patients with favorable cytogenetic. No significant difference for DFS or OS was found in other comparisons.

### Network meta-analysis

**Figure 3:**
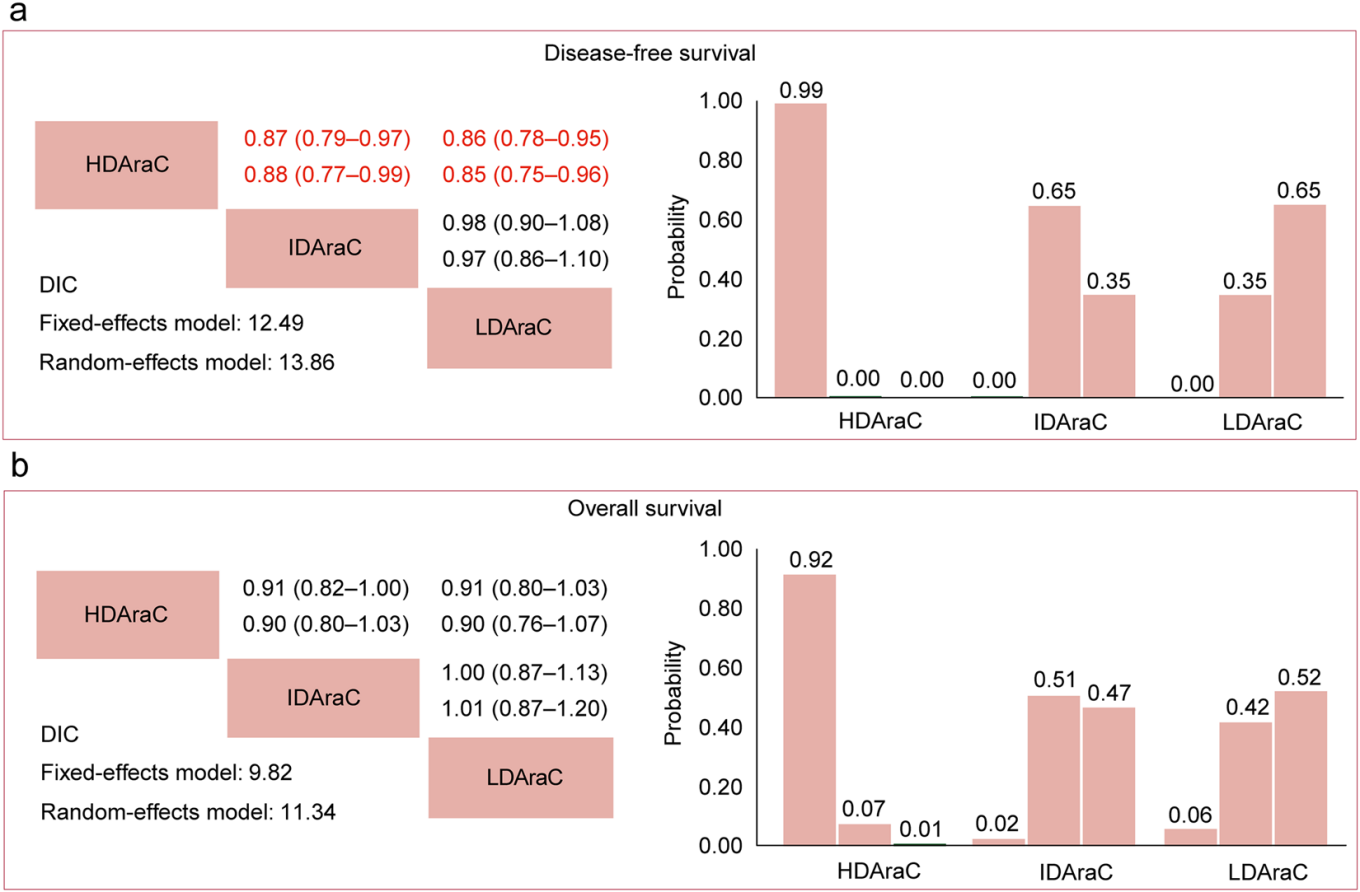
Network meta-analysis for disease-free survival (**a**) and overall survival (**b**). Upper triangles denote pooled hazard ratios (HRs). The column dose range is compared with the row dose range. In each cell, the first and second line used fixed-effect and random-effect model. Numbers in parentheses indicate 95% credible intervals. HRs with Bayesian *p* value < 0.05 are in red. Lower triangles denote the Bayesian deviance information criterion (DIC) statistics from the fixed- and random-effects models. Cumulative probabilities of each dose range ranking first, second and third best based on the corresponding effect-model with lower DIC values.

The network comparisons consisted of the three dose ranges of Ara-C (Supplementary Fig. S9). Both fixed and random effect models were reported; but the effect models with relatively lower DIC values, indicating relatively lower heterogeneity across trials and simpler models, were chosen for summary estimation. In the comparisons across all cytogenetics (Fig. 3a and b), HDAraC in consolidation significantly improved DFS compared with either IDAraC (HR 0.87, 95% CrI 0.79–0.97) or LDAraC (HR 0.86, 95% CrI 0.78–0.95). No significant difference for OS was found in all comparisons. In ranking of the three Ara-C dose ranges (Fig. 3), the cumulative probabilities of being the most efficacious dose in consolidation chemotherapy were as follows (DFS, OS): HDAraC (99%, 92%), IDAraC (0%, 2%), LDAraC (0%, 6%).

**Figure 4:**
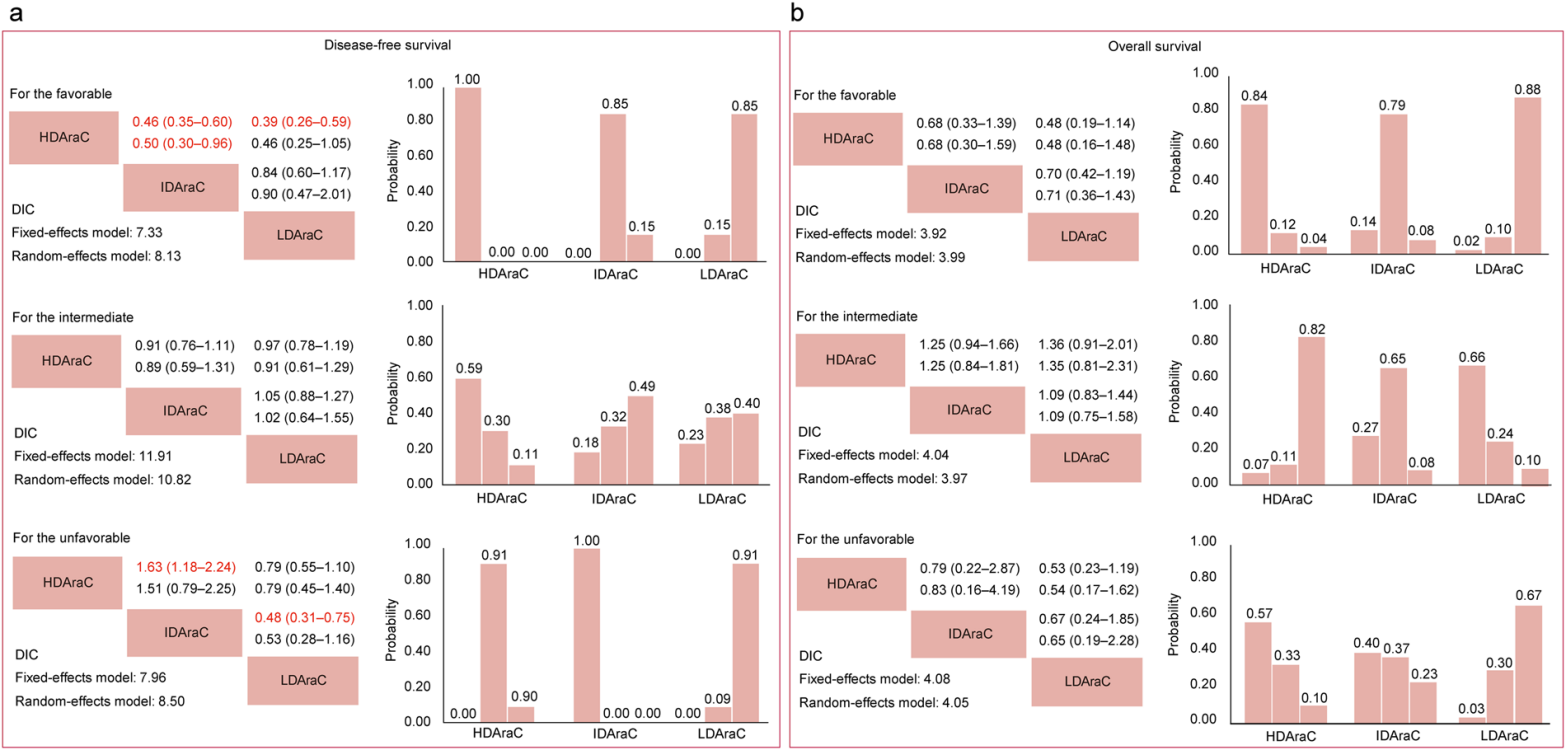
Network meta-analysis for disease-free survival (**a**) and overall survival (**b**) in patients stratified by cytogenetic risk groups. Upper triangles denote pooled hazard ratios (HRs). The column dose range is compared with the row dose range. In each cell, the first and second line used fixed-effect and random-effect model. Numbers in parentheses indicate 95% credible intervals. HRs with Bayesian *p* value < 0.05 are in red. Lower triangles denote the Bayesian deviance information criterion (DIC) statistics from the fixed- and random-effects models. Cumulative probabilities of each dose range ranking first, second and third best based on the corresponding effect-model with lower DIC values.

In subgroup analysis stratified by cytogenetics (Fig. 4a and b), HDAraC in consolidation chemotherapy significantly benefited DFS for patients with favorable cytogenetic compared with IDAraC (HR 0.46, 95% CrI 0.35–0.60) and LDAraC (HR 0.39, 95% CrI 0.26–0.59). For the unfavorable ones, however, IDAraC provided a DFS benefit over HDAraC and LDAraC. In the ranking the three Ara-C dose ranges for different cytogenetics, the cumulative probabilities of being the most efficacious dose of Ara-C in consolidation chemotherapy for DFS were as follows (favorable, intermediate, unfavorable): HDAraC (100%, 59%, 0%), IDAraC (0%, 18%, 100%), LDAraC (0%, 23%, 0%). No significant difference for OS was found in subgroup analysis.

**Figure 5:**
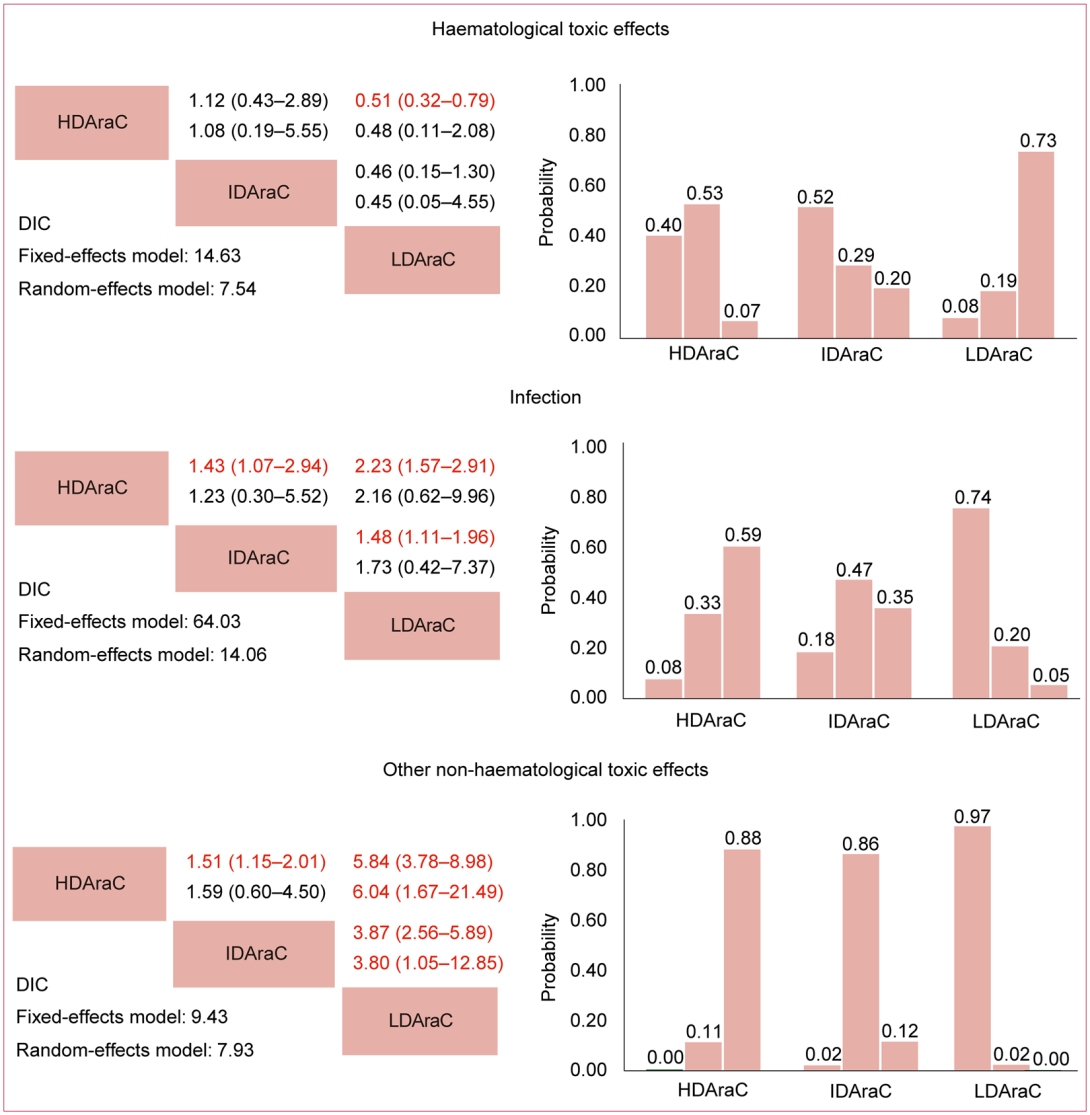
Network meta-analysis for haematological toxic effects, infection and other non-haematological toxic effects. Upper triangles denote pooled hazard ratios (ORs). The column dose range is compared with the row dose range. In each cell, the first and second line used fixed-effect and random-effect model. Numbers in parentheses indicate 95% credible intervals. HRs with Bayesian *p* value < 0.05 are in red. Lower triangles denote the Bayesian deviance information criterion (DIC) statistics from the fixed- and random-effects models. Cumulative probabilities of each dose range ranking first, second and third best based on the corresponding effect-models with lower DIC values.

ORs for haematological toxic effects, infection and other non-haematological toxic effects could be respectively estimated in four, seven and four trials. Four studies did not report the overall number of haematological toxic effects, but separately reported the number of grade 3–4 leukopenia, thrombocytopenia and neutropenia. We used the largest of the three numbers to calculate the trial-specific ORs for haematological toxic effects^8, 10, 12, 18^. Four studies did not report the overall number of non–haematological toxic effects, but separately reported the number of individual non–haematological toxic reactions, and we used their sum to calculate the trial-specific ORs for non-haematological toxic effects^8, 11, 12, 18^. Two studies separately reported toxic effects in each course, but not the overall number during consolidation therapy, and we used the largest number to estimate to calculate the trial-specific ORs^8, 11^. Network comparisons of grade 3–4 toxic effects were presented in Fig. 5. No significant difference was found for haematological toxic effects or infection among different doses of Ara-C. For other non-haematological toxic effects, when compared with LDAraC, HDAraC (HR 6.04, 95% CrI 3.78–8.98) and IDAraC (HR 3.80, 95% CrI 1.05–12.85) were associated with higher risk of incidences. No significant difference between HDAraC and IDAraC was found.

In all network comparisons, no significant inconsistency was indicated in node-splitting analysis.

### Sensitivity analysis

Sensitivity analysis showed that excluding the three studies using the non-conventional Ara-C doses in induction did not alter the overall effect size for DFS across all cytogenetics (Supplementary Table S1, Figs S2 and S3). In addition, HDAraC further showed an OS benefit when compared with IDAraC (HR 0.85, 95% CrI 0.73–0.98) and LDAraC (HR 0.86, 95% CrI 0.74–1.00) in this sensitivity analysis.

Further sensitivity analysis dividing AML201 into HDAraC-used trials did not alter the overall effect size for DFS or OS across all cytogenetics (Supplementary Table S2, Figs S4 and S5).

## Discussion

A variety of strategies to prevent relapse for AML patients have been explored for over 30 years. One of the most notable progress is the standard post-remission chemotherapy for adult AML patients established based on the CALGB 8525 protocol: single-agent high-dose Ara-C in a dosage of 2–3 g/m^2^ twice daily on days 1, 3, 5 for at least two cycles^4–6^. Given the toxicity and high price of HDAraC, numerous randomized trials were conducted for exploration of dosage de-escalation^7, 8, 10–12, 15^. However, most of them comparing IDAraC or LDAraC (usually in combination with other drugs) with “CALGB style” HDAraC failed to show a significant improvement in any survival endpoints. On the contrary, evidences tended to favor HDAraC in some trials: in Medical Research Council (MRC) AML 15 trial, halving dosage from 3 g/m^2^ to 1.5 g/m^2^ was associated with a strong trend towards a higher cumulative incidence of relapse^11^; and a per protocol analysis in SAL AML 2003 trial showed an OS advantage in the single-agent HDAraC group^10^. With evaluating these individual trials, there are two different opinions on Ara-C dosage in consolidation for adult AML patients: (1) In consideration of the comparable therapeutic effect and less toxicity, the IDAraC in a dosage of 1–1.5 g/m^2^ over 3 days with a cumulative dose of 6–18 g should be recommended to be a new standard^19^. (2) Due to lack of important and consistent improvements in outcome from existing evidences, HDAraC in a dosage of 2–3 g/m^2^ over 3 days remains the standard for post-remission chemotherapy^5^. Nowadays, judgements on the standard post-remission chemotherapy do not reach consensus and the optimal dose of Ara-C remains unclear. Therefore, a network meta-analysis is needed to address this issue.

To the best of our knowledge, this is the first meta-analysis assessing the benefit and toxicity for different doses of Ara-C. Our results show that HDAraC in a dosage of 3 g/m^2^ twice daily in consolidation chemotherapy can significantly prolong DFS by at least 13% when compared with lower-dose Ara-C (≤2 g/m^2^ twice daily) for adult AML patients; and this advantage is focused on the patients with favorable cytogenetics, but not the other cytogenetics. Among the ten trials of our meta-analysis, SAL AML 96, Acute Leukemia French Association (ALFA) 9802 and Australasian Leukaemia and Lymphoma Group (ALLG) M7 trial used non-conventional doses of Ara-C which were distinct from others in induction therapy, so we did sensitivity analysis by excluding these three trials. Further, in Japan Adult Leukemia Study Group (JALSG) AML 201 trial, a single dose of 2 g/m² twice daily in consolidation was divided into LDAraC. However, relatively more frequent injections of Ara-C in this trial resulted in a cumulative dose of 60 g, which belonged to high cumulative dose range. We thus did sensitivity analysis by re-dividing this trial into HDAraC. In addition, we also made comparisons for the younger adults aged <65 in a sensitivity analysis (Supplementary Figs S6–S8). All the three sensitivity analysis did not alter the DFS benefit of HDAraC.

In this meta-analysis, we found that HDAraC and IDAraC in consolidation chemotherapy were associated with higher risk of grade 3–4 non-haematological toxic effects when compared with LDAraC. However, importantly, we noticed no significant difference between HDAraC and IDAraC in terms of both grade 3–4 haematological and non-haematological toxic effects.

Our study has some advantages and important suggestions. First, rather than only comparing HDAraC with IDAraC or LDAraC in individual trials, our study included all the comparable randomized trials using different doses of Ara-C in consolidation within a single meta-analysis and compared these dosages simultaneously, achieving greater statistical power and avoiding potential selection bias. Second, RCTs included were multicenter, randomized phase III/IV trials performed at the national level by cooperative study groups, and these trials with generally high quality ensures reliability of the analysis results. Third, using Bayesian network methods, we compared dosages indirectly when head-to-head comparisons were insufficient and obtained precise estimates of effect by jointly evaluating direct and indirect comparisons. Fourth, we did several sensitivity analysis to test the robustness of results and the conclusion remains valid. Our synthesis of existing evidence provides useful information on clinical value of HDAraC, which should be reconsidered in clinical care and future research.

Potential limitations of our study should be noted. First, like most of the published meta-analysis, our analysis is based on the summary data from published literature rather than individual patient data, which limit the detail that can be captured regarding subgroups. We could not evaluate outcomes for clinically relevant subgroups other than cytogenetic risk. Therefore, our findings need to be considered as average effects. Second, there are few trials purely comparing different doses of Ara-C without other chemotherapeutic agents in consolidation^7, 11^. The impact on other chemotherapeutic agents in our study could not be completely eliminated. As Ara-C is till now the most active compound in consolidation therapy, we believe that the other relevant agents performed in included RCTs played complementary roles in Ara-C based therapy. Thus, our estimates remain effective. Third, the reporting of toxic effects was incomplete and inconsistent in included trials, and thus we had to use imputed data as described in our results. Our meta-analysis on toxicity should be interpreted with some caution.

In conclusion, our meta-analysis shows that Ara-C in a dosage of 3 g/m^2^ twice daily provides maximal therapeutic effect in consolidation chemotherapy for adult AML patients. Though it is associated with grade 3–4 non-haematological toxicity compared with low-dose Ara-C in a dosage <1 g/m^2^, the toxic difference between the doses of 3 g/m^2^ and 1–2 g/m^2^ is non-significant.

## Methods

This study was reported according to preferred reporting items for systematic reviews and meta-analysis (PRISMA) guidelines.

### Ethics approval and consent to participate

Ethics approval for this network meta-analysis was not required.

### Literature search and study selection

We underwent searches of PubMed, the Cochrane database and Embase, combing the search terms “cytarabine”; acut* and leukem*/leukaem*/leucem*/leucaem*/aml; myelo* or nonlympho* from January 1994 to June 2016 without language restriction. Two independent reviewers (W.D. and C.D.) conducted study selection based on the “PICOS” criteria (i.e., Patient, Intervention, Comparator, Outcome, Study design):

- P: Adults aged 15 years or older and have newly diagnosed acute myeloid leukaemia (either de novo or secondary) or high-risk myelodysplastic syndrome.
- I and C: Different doses of Ara-C performed in two or more arms in consolidation.
- O: Disease-free survival, overall survival and grade 3–4 toxic effects.
- S: Randomized controlled trials.

The trials that included only patients with acute promyelocytic leukaemia were excluded. We also searched for additional trials in the reference list of relevant reviews, meta-analysis and bibliographies in the discipline. Only the most updated or most inclusive data for a given study was included.

### Data extraction and risk of bias assessment

Two reviewers (W.D. and C.D.) separately recorded trial design, entry criteria, patient characteristics, adequacy of induction therapy (regimens performed and percentage of patients achieved CR), Ara-C treatment in consolidation randomization, cumulative dose of Ara-C per course, follow-up and outcomes (disease-free survival, overall survival, grade 3–4 haematological and non-haematological toxic effects).

Risk of bias of individual trials were assessed independently by the same reviewers with the Cochrane risk of bias tool^20^. Conflicts were resolved by consensus.

### Statistical analysis

The primary outcome in our study was disease-free survival (DFS). Secondary pre-specified endpoints included overall survival (OS), treatment-related grade 3–4 haematological, infection and other non-haematological toxic effects. These outcomes were defined in accordance with the revised International Working Group criteria for the therapeutic trials in AML^21^. We measured hazard ratios (HRs) for time-to-event outcomes (DFS and OS) and odds ratio (ORs) for dichotomous data (grade 3–4 toxic effects). When HRs were not explicitly provided, we estimated them according to the method detailed by Tierney and colleagues^22^.

Two types of meta-analysis were conducted. First, standard pairwise comparisons were built with STATA 12.0 (STATA Crop., College Station, TX, USA). Both fixed and random effect models were reported. In all the comparisons, we used fixed effect models if the heterogeneity across trials was not significant (a *P* value < 0.10 in *χ* ^2^ test or an *I* ^*2*^ < 50% in *I* ^2^ metric); otherwise, we explored the heterogeneity and the random effect models were used^23^. Second, mixed network comparisons were built with WinBUGS 1.4.3 (MRC Biostatistics Unit, Cambridge, UK), allowing for the combination of direct and indirect evidence into a combined overall point estimate. Treatment effects were estimated by posterior means with corresponding 95% credible intervals (CrIs), which are the Bayesian analog of the 95% confidence intervals (CIs)^24^. Both fixed and random effect models were applied with non-informative uniform and normal prior distributions, yielding 50,000 iterations with a burn-in number of 10,000 iterations and a thin interval of 50 to obtain the posterior distributions of the model parameters^25^. Then the deviance information criterion (DIC) statistics were used to compare the two models: the effect model with relatively lower DIC value indicated lower heterogeneity across trials and a simpler model, and the corresponding results were chosen for summary estimation^26^. Convergence of iterations was evaluated according to Gelman-Rubin-Brooks statistic^27^. The probability of each treatment in the ranking was evaluated based on its posterior probabilities, which depended on counting the proportion of iterations in the Markov chain of HR or OR ranking in the treatments^28, 29^. Results from network meta-analysis were compared with standard pairwise meta-analysis to evaluate whether there was inconsistency. Node-splitting analysis was also applied to evaluate inconsistency for closed loops in the network^30, 31^. Significant inconsistency was indicated if node-splitting analysis derived *P* < 0.05 of disagreement between direct and indirect evidence.

In subgroup analysis, we assessed DFS and OS benefit for cytogenetic risk subgroups: the favorable, intermediate and unfavorable cytogenetic risk patients, which were classified by cytogenetic abnormalities^32, 33^. The robustness of main findings was further tested by additional sensitivity analysis. We looked for systematic difference in induction and consolidation strategies.

Publication bias could not be formally evaluated because of the small number of studies included in each direct comparison. Although the potential for this bias is real given the small number of studies and the for-profit interest, we judged that this concern was not likely to decrease certainty in the evidence.

## Electronic supplementary material

Supplementary Information

## References

[1] Burnett A, Wetzler M, Lowenberg B (2011). Therapeutic Advances in Acute Myeloid Leukemia. J Clin Oncol.

[2] Dohner H (2010). Diagnosis and management of acute myeloid leukemia in adults: recommendations from an international expert panel, on behalf of the European LeukemiaNet. Blood.

[3] Dohner H, Weisdorf DJ, Bloomfield CD (2015). Acute Myeloid Leukemia. N Engl J Med.

[4] Rowe JM (2008). Consolidation therapy: What should be the standard of care?. Best Pract Res Clin Haematol.

[5] Schlenk RF (2014). Post-remission therapy for acute myeloid leukemia. Haematologica.

[6] De Kouchkovsky I, Abdul-Hay M (2016). ‘Acute myeloid leukemia: a comprehensive review and 2016 update’. Blood Cancer J.

[7] Mayer RJ (1994). Intensive postremission chemotherapy in adults with acute myeloid leukemia. N Engl J Med.

[8] Thomas X (2011). Comparison of high-dose cytarabine and timed-sequential chemotherapy as consolidation for younger adults with AML in first remission: the ALFA-9802 study. Blood.

[9] Miyawaki S (2011). A randomized comparison of 4 courses of standard-dose multiagent chemotherapy versus 3 courses of high-dose cytarabine alone in postremission therapy for acute myeloid leukemia in adults: the JALSGAML201 Study. Blood.

[10] Schaich M (2013). High-Dose Cytarabine Consolidation With or Without Additional Amsacrine and Mitoxantrone in Acute Myeloid Leukemia: Results of the Prospective Randomized AML2003 Trial. J Clin Oncol.

[11] Burnett AK (2013). Optimization of chemotherapy for younger patients with acute myeloid leukemia: results of the medical research council AML15 trial. J Clin Oncol.

[12] Schaich M (2011). Cytarabine Dose of 36 g/m^2^ Compared With 12 g/m^2^ Within First Consolidation in Acute Myeloid Leukemia: Results of Patients Enrolled Onto the Prospective Randomized AML96 Study. J Clin Oncol.

[13] Ferrara F, Schiffer CA (2013). Acute myeloid leukaemia in adults. The Lancet.

[14] Saultz JN, Garzon R (2016). Acute Myeloid Leukemia: A Concise Review. J Clin Med.

[15] Weick JK (1996). A randomized investigation of high-dose versus standard-dose cytosine arabinoside with daunorubicin in patients with previously untreated acute myeloid leukemia: A Southwest Oncology Group study. Blood.

[16] Zittoun RA (1995). Autologous or allogeneic bone marrow transplantation compared with intensive chemotherapy in acute myelogenous leukemia. N Engl J Med.

[17] Fopp M (1997). Post-remission therapy of adult acute myeloid leukaemia: One cycle of high-dose versus standard-dose cytarabine. Annals of oncology.

[18] Bradstock KF (2005). A randomized trial of high- versus conventional-dose cytarabine in consolidation chemotherapy for adult de novo acute myeloid leukemia in first remission after induction therapy containing high-dose cytarabine. Blood.

[19] Lowenberg B (2013). Sense and nonsense of high-dose cytarabine for acute myeloid leukemia. Blood.

[20] Higgins JP (2011). The Cochrane Collaboration’s tool for assessing risk of bias in randomised trials. BMJ.

[21] Cheson BD (2003). Revised recommendations of the International Working Group for Diagnosis, Standardization of Response Criteria, Treatment Outcomes, and Reporting Standards for Therapeutic Trials in Acute Myeloid Leukemia. J Clin Oncol.

[22] Tierney JF, Stewart LA, Ghersi D, Burdett S, Sydes MR (2007). Practical methods for incorporating summary time-to-event data into meta-analysis. Trials.

[23] Higgins, J. P. T. & Green, S. *Cochrane Handbook version 5.1.0*., (March, 2011. http://handbook.cochrane.org. (Accessed April 9, 2015).

[24] Wandel S (2010). Effects of glucosamine, chondroitin, or placebo in patients with osteoarthritis of hip or knee: network meta-analysis. BMJ.

[25] Woods BS, Hawkins N, Scott DA (2010). Network meta-analysis on the log-hazard scale, combining count and hazard ratio statistics accounting for multi-arm trials: a tutorial. BMC Med Res Methodol.

[26] Spiegelhalter DJ, Best NG, Carlin BP, Linde AVD (2002). Bayesian measures of model complexity and fit. J R Stat Soc.

[27] Brooks SP, Gelman A (1998). General Methods for Monitoring Convergence of Iterative Simulations. Journal of Computational and Graphical Statistics.

[28] Mills EJ, Thorlund K, Ioannidis JP (2013). Demystifying trial networks and network meta-analysis. BMJ.

[29] Salanti G, Ades AE, Ioannidis JP (2011). Graphical methods and numerical summaries for presenting results from multiple-treatment meta-analysis: an overview and tutorial. J Clin Epidemiol.

[30] Higgins JP (2012). Consistency and inconsistency in network meta-analysis: concepts and models for multi-arm studies. Res Synth Methods.

[31] Dias S, Welton NJ, Caldwell DM, Ades AE (2010). Checking consistency in mixed treatment comparison meta-analysis. Stat Med.

[32] Schoch C, Haferlach T (2002). Cytogenetics in acute myeloid leukemia. CURR ONCOL REP.

[33] Walker H, Smith F, Betts D (1994). Cytogenetics in acute myeloid leukaemia. BLOOD REV.

