# Supplementary material for: Efficacy and safety of different doses of cytarabine in consolidation therapy for adult acute myeloid leukemia patients: a network meta-analysis
Source: Sci Rep. 2017 Aug 25;7:9509. doi: 10.1038/s41598-017-10368-0 (PMC5572788; doi:10.1038/s41598-017-10368-0)
Supplement: Supplementary file 1 — Supplementary Information [file 41598_2017_10368_MOESM1_ESM.doc]

**Supplementary Information**

Efficacy and safety of different doses of cytarabine in consolidation therapy for adult acute myeloid leukemia patients: a network meta-analysis

Di Wu^1, *^ , Chongyang Duan^2, *^ , Liyong Chen^1^ & Size Chen^3^

**Fig. S1** Cochrane risk of bias tool assessment. Given the unambiguous study treatments and “strict” endpoints (disease-free survival and overall survival), we did not anticipate any impact of lack of blinding on outcomes. For treatment-related toxicity, all trials used pre-planned standard grading methods and uniform follow-up scheme for all arms.

**Fig. S2** Direct meta-analysis for disease-free survival and overall survival after excluding AML M7, ALFA9802 and AML96. (a) and (b) All patients. (c) According to cytogenetic risk groups. The size of the boxes is proportional to the amount of data contained in each data line. The bars indicate 95% confidence intervals (CIs). HDAraC, high–dose cytarabine (> 2 g/m^2^, ≤ 3 g/m^2^ twice daily); IDAraC, intermediate–dose cytarabine (≥ 1 g/m2, ≤ 2 g/m^2^ twice daily); LDAraC, low–dose cytarabine (< 1 g/m^2^ twice daily); I–V = inverse variance. D+L = DerSimonan and Laird.

**Fig. S3** Network meta-analysis for disease-free survival and overall survival after excluding AML M7, ALFA9802 and AML96. (a) All patients. (b) According to cytogenetic risk groups. Upper triangles denote pooled hazard ratios (HRs). The column dose range is compared with the row dose range. In each cell, the first and second line used fixed-effect and random-effect model. Numbers in parentheses indicate 95% credible intervals. HRs with Bayesian *p* value < 0.05 are in red. Lower triangles denote the Bayesian deviance information criterion (DIC) statistics from the fixed- and random-effects models. Cumulative probabilities of each dose range ranking first, second and third best based on the corresponding effect-model with lower DIC values. HDAraC, high–dose cytarabine (> 2 g/m^2^, ≤ 3 g/m^2^ twice daily); IDAraC, intermediate–dose cytarabine (≥ 1 g/m2, ≤ 2 g/m^2^ twice daily); LDAraC, low–dose cytarabine (< 1 g/m^2^ twice daily); I–V = inverse variance. D+L = DerSimonan and Laird.

**Fig. S4** Direct meta-analysis for disease-free survival and overall survival after dividing AML201 into high-dose cytarabine-used trials. (a) and (b) All patients. (c) and (d) According to cytogenetic risk groups. The size of the boxes is proportional to the amount of data contained in each data line. The bars indicate 95% confidence intervals (CIs). I–V = inverse variance. D+L = DerSimonan and Laird. The bars indicate 95% confidence intervals (CIs). HDAraC, high–dose cytarabine (> 2 g/m^2^, ≤ 3 g/m^2^ twice daily); IDAraC, intermediate–dose cytarabine (≥ 1 g/m2, ≤ 2 g/m^2^ twice daily); LDAraC, low–dose cytarabine (< 1 g/m^2^ twice daily); I–V = inverse variance. D+L = DerSimonan and Laird.

**Fig. S5** Network meta-analysis for disease-free survival and overall survival after dividing AML201 into high-dose cytarabine-used trials. (a) and (b) All patients. (c) and (d) According to cytogenetic risk groups. Upper triangles denote pooled hazard ratios (HRs). The column dose range is compared with the row dose range. In each cell, the first and second line used fixed-effect and random-effect model. Numbers in parentheses indicate 95% credible intervals. HRs with Bayesian *p* value < 0.05 are in red. Lower triangles denote the Bayesian deviance information criterion (DIC) statistics from the fixed- and random-effects models. Cumulative probabilities of each dose range ranking first, second and third best based on the corresponding effect-model with lower DIC values. HDAraC, high–dose cytarabine (> 2 g/m^2^, ≤ 3 g/m^2^ twice daily); IDAraC, intermediate–dose cytarabine (≥ 1 g/m2, ≤ 2 g/m^2^ twice daily); LDAraC, low–dose cytarabine (< 1 g/m^2^ twice daily); I–V = inverse variance. D+L = DerSimonan and Laird.

**Fig. S6** Network meta-analysis results for disease-free survival (a) and overall survival (b) for patients < 65 years. Upper triangles denote pooled hazard ratios (HRs). The column dose range is compared with the row dose range. In each cell, the first and second line used fixed-effect and random-effect model. Numbers in parentheses indicate 95% credible intervals. HRs with Bayesian *p* value < 0.05 are in red. Lower triangles denote the Bayesian deviance information criterion (DIC) statistics from the fixed- and random-effects models. Cumulative probabilities of each dose range ranking first, second and third best were based on the fixed-effect model. HDAraC, high–dose cytarabine (> 2 g/m^2^, ≤ 3 g/m^2^ twice daily); IDAraC, intermediate–dose cytarabine (≥ 1 g/m2, ≤ 2 g/m^2^ twice daily); LDAraC, low–dose cytarabine (< 1 g/m^2^ twice daily); I–V = inverse variance. D+L = DerSimonan and Laird.

**Fig. S7** Network meta-analysis results for disease-free survival (a) and overall survival (b) for patients < 65 years after excluding AML M7, ALFA9802 and AML96. Upper triangles denote pooled hazard ratios (HRs). The column dose range is compared with the row dose range. In each cell, the first and second line used fixed-effect and random-effect model. Numbers in parentheses indicate 95% credible intervals. HRs with Bayesian *p* value < 0.05 are in red. Lower triangles denote the Bayesian deviance information criterion (DIC) statistics from the fixed- and random-effects models. Cumulative probabilities of each dose range ranking first, second and third best were based on the fixed-effect model. HDAraC, high–dose cytarabine (> 2 g/m^2^, ≤ 3 g/m^2^ twice daily); IDAraC, intermediate–dose cytarabine (≥ 1 g/m2, ≤ 2 g/m^2^ twice daily); LDAraC, low–dose cytarabine (< 1 g/m^2^ twice daily); I–V = inverse variance. D+L = DerSimonan and Laird.

**Fig. S8** Network meta-analysis results for disease-free survival (a) and overall survival (b) for patients < 65 years after dividing AML201 into high-dose cytarabine-used trials. Upper triangles denote pooled hazard ratios (HRs). The column dose range is compared with the row dose range. In each cell, the first and second line used fixed-effect and random-effect model. Numbers in parentheses indicate 95% credible intervals. HRs with Bayesian *p* value < 0.05 are in red. Lower triangles denote the Bayesian deviance information criterion (DIC) statistics from the fixed- and random-effects models. Cumulative probabilities of each dose range ranking first, second and third best were based on the fixed-effect model. HDAraC, high–dose cytarabine (> 2 g/m^2^, ≤ 3 g/m^2^ twice daily); IDAraC, intermediate–dose cytarabine (≥ 1 g/m2, ≤ 2 g/m^2^ twice daily); LDAraC, low–dose cytarabine (< 1 g/m^2^ twice daily); I–V = inverse variance. D+L = DerSimonan and Laird.

**Fig. S9** Multiple treatment comparison network diagram. The size of the nodes is proportional to the number of patients (in parentheses) randomized to receive the treatment. The width of the lines is proportional to the number of trials (beside the line) comparing the connected treatments. DFS, disease-free survival; HDAraC, high-dose cytarabine (> 2 g/m^2^, ≤ 3 g/m^2^ twice daily); IDAraC, intermediate-dose cytarabine (≥ 1 g/m^2^, ≤ 2 g/m^2^ twice daily); LDAraC, low-dose cytarabine (< 1 g/m^2^ twice daily); OS, overall survival.


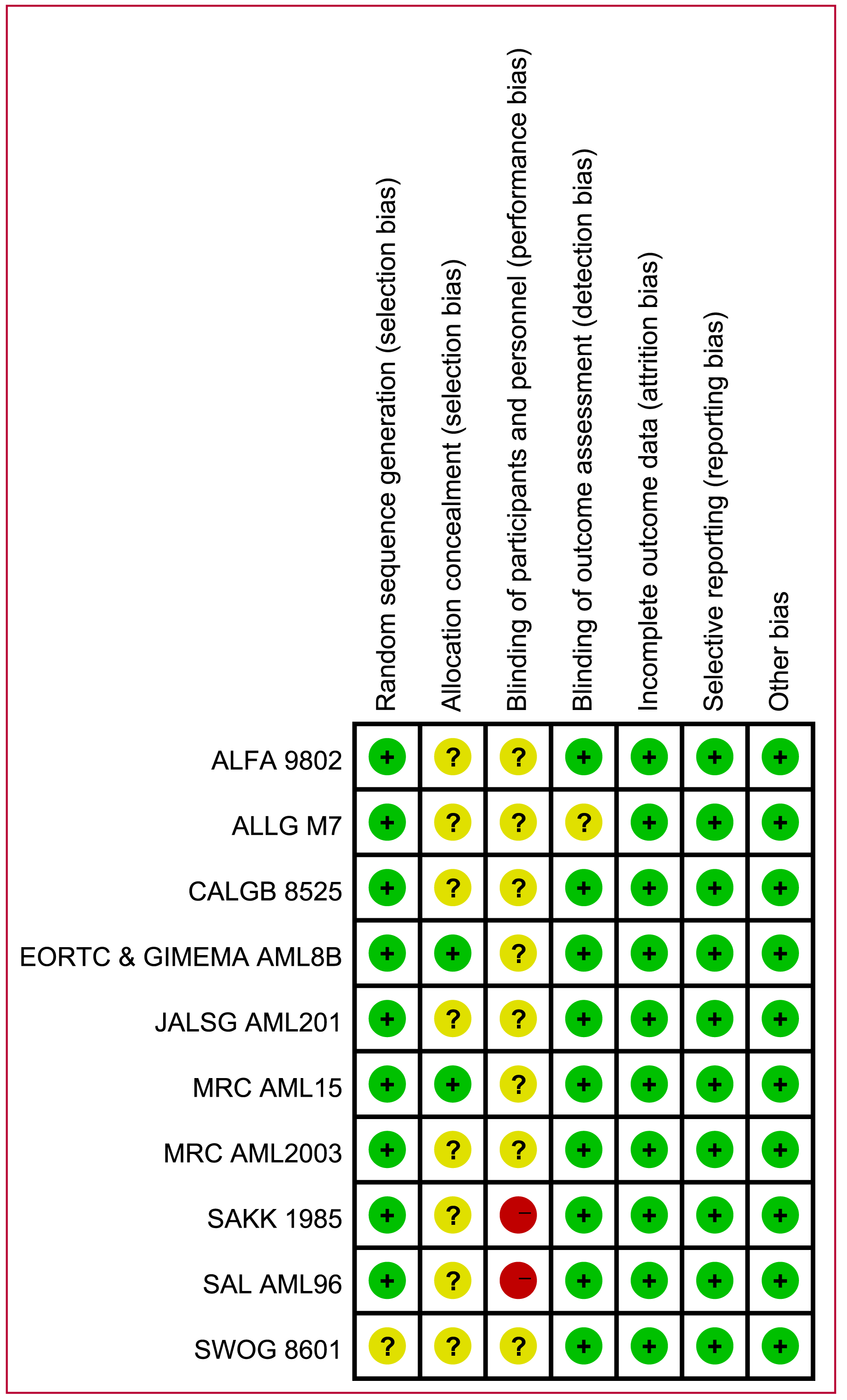
**Fig. S1**

**Fig. S2**

**
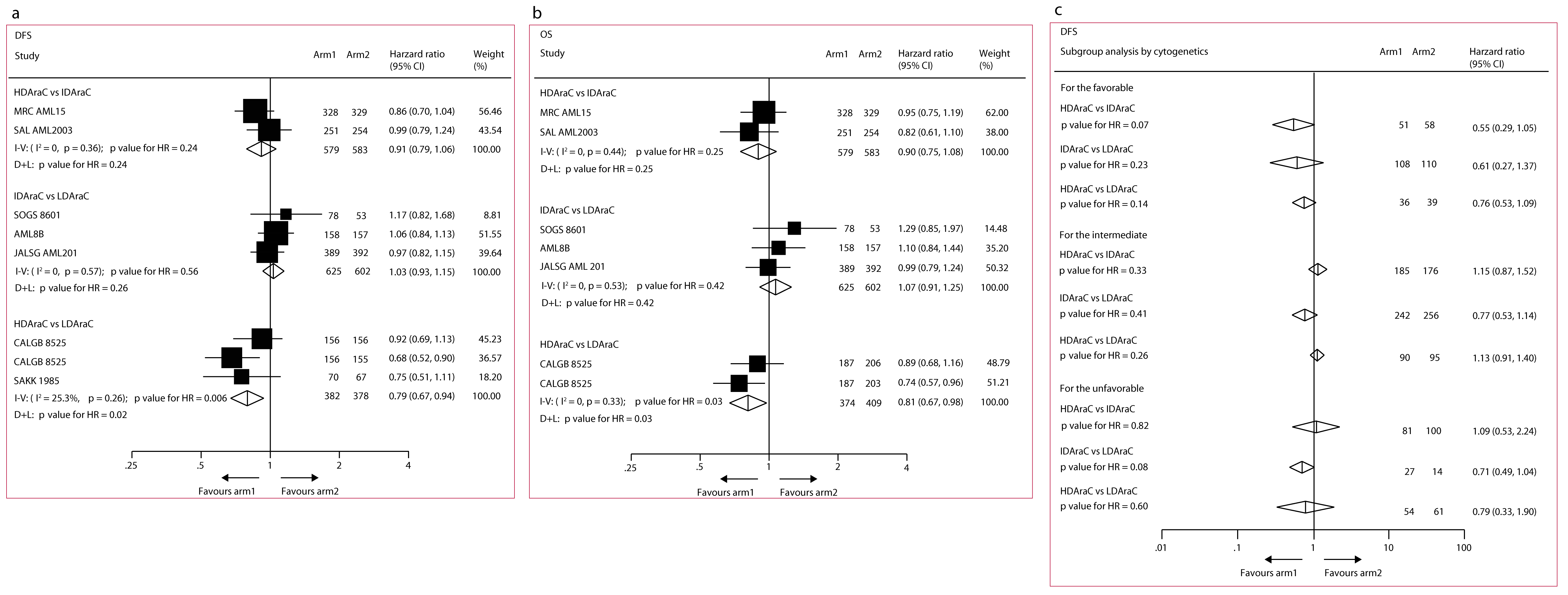
**


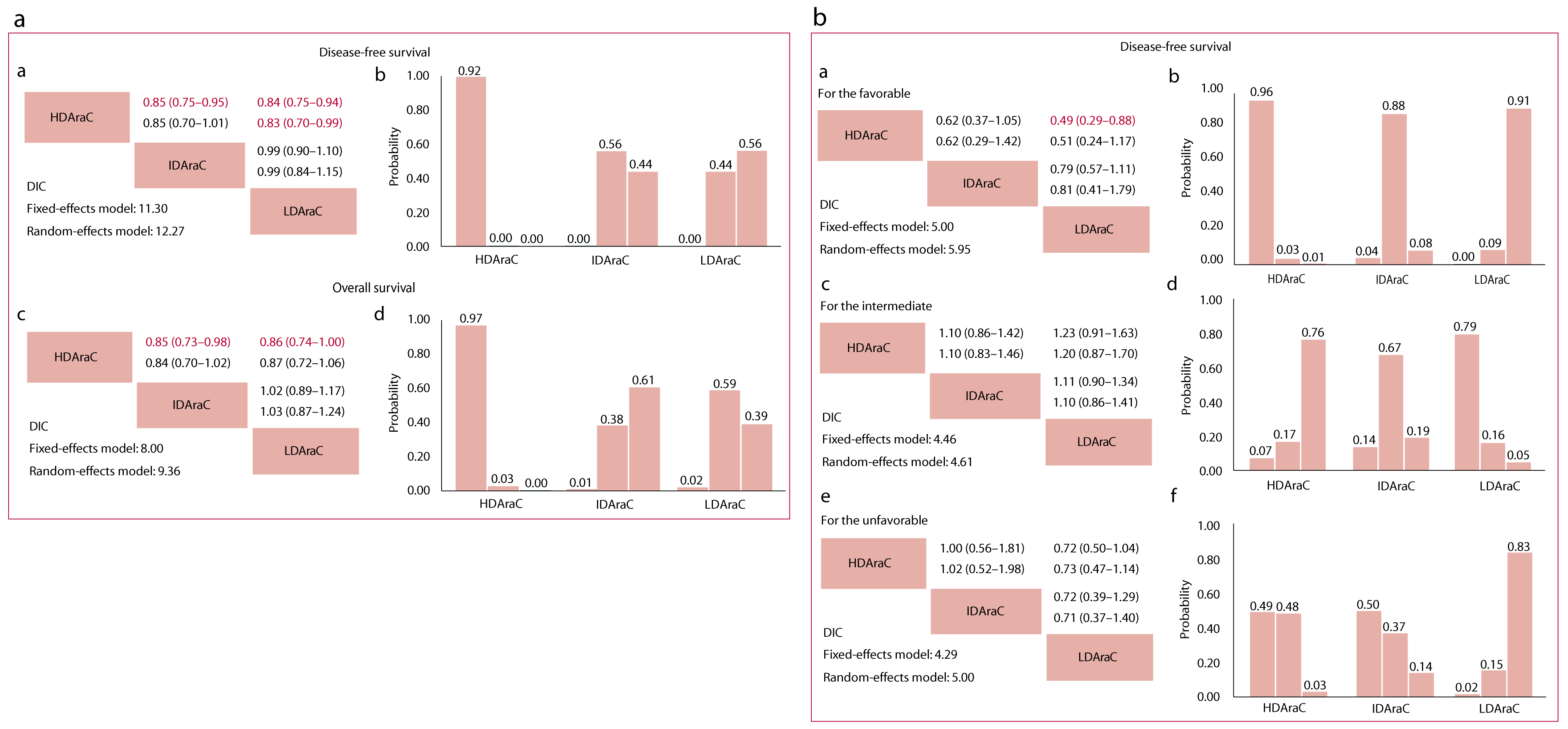
 **Fig. S3**


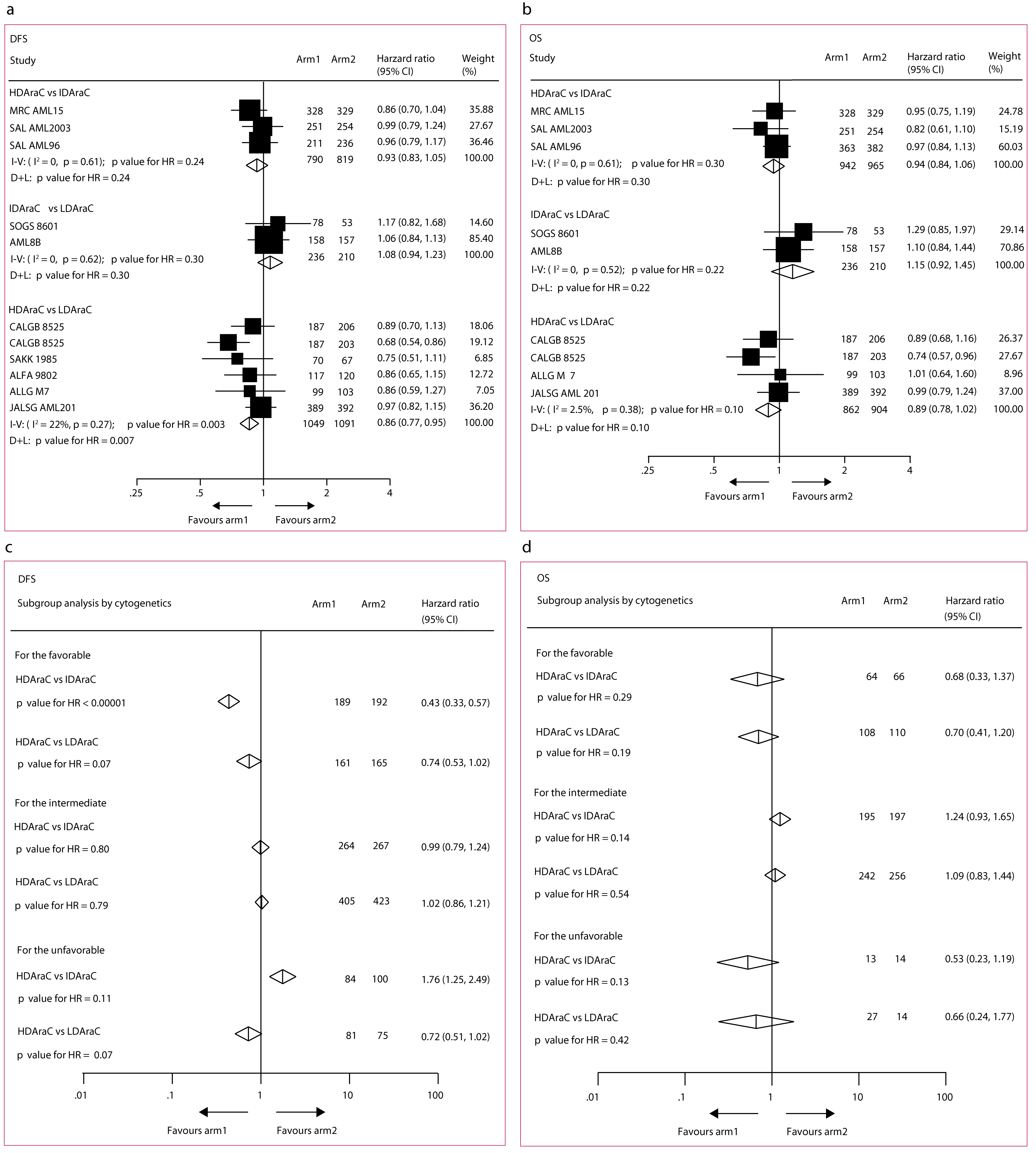
**Fig. S4**

**Fig. S5**

**
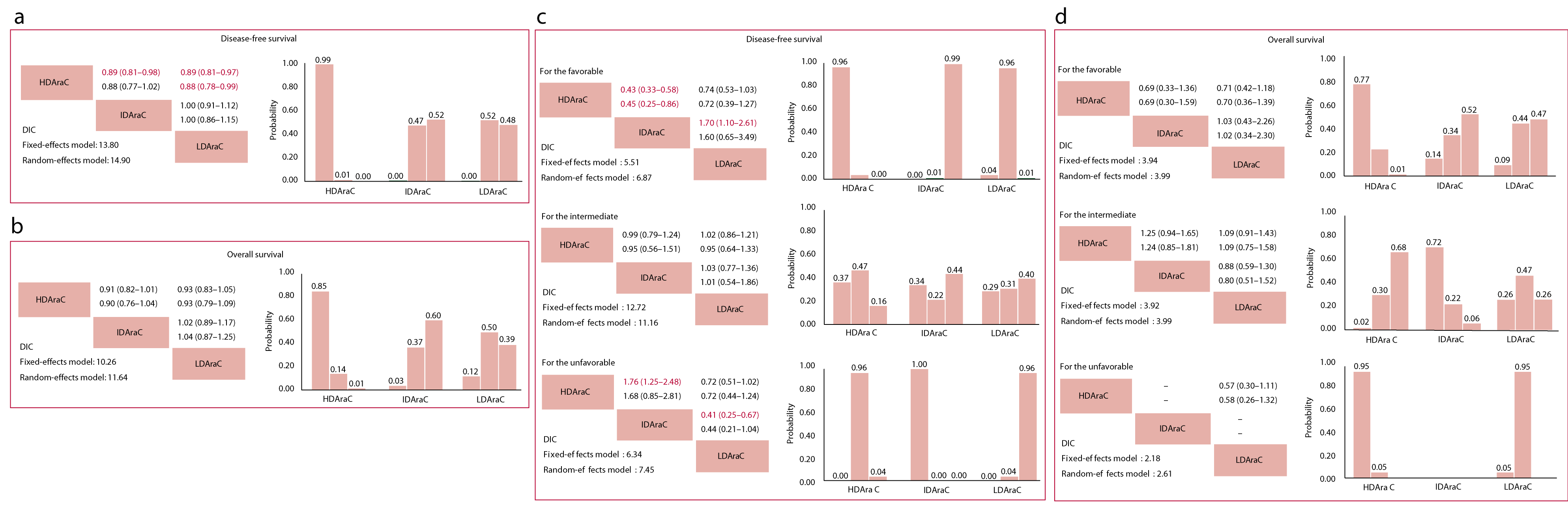
**


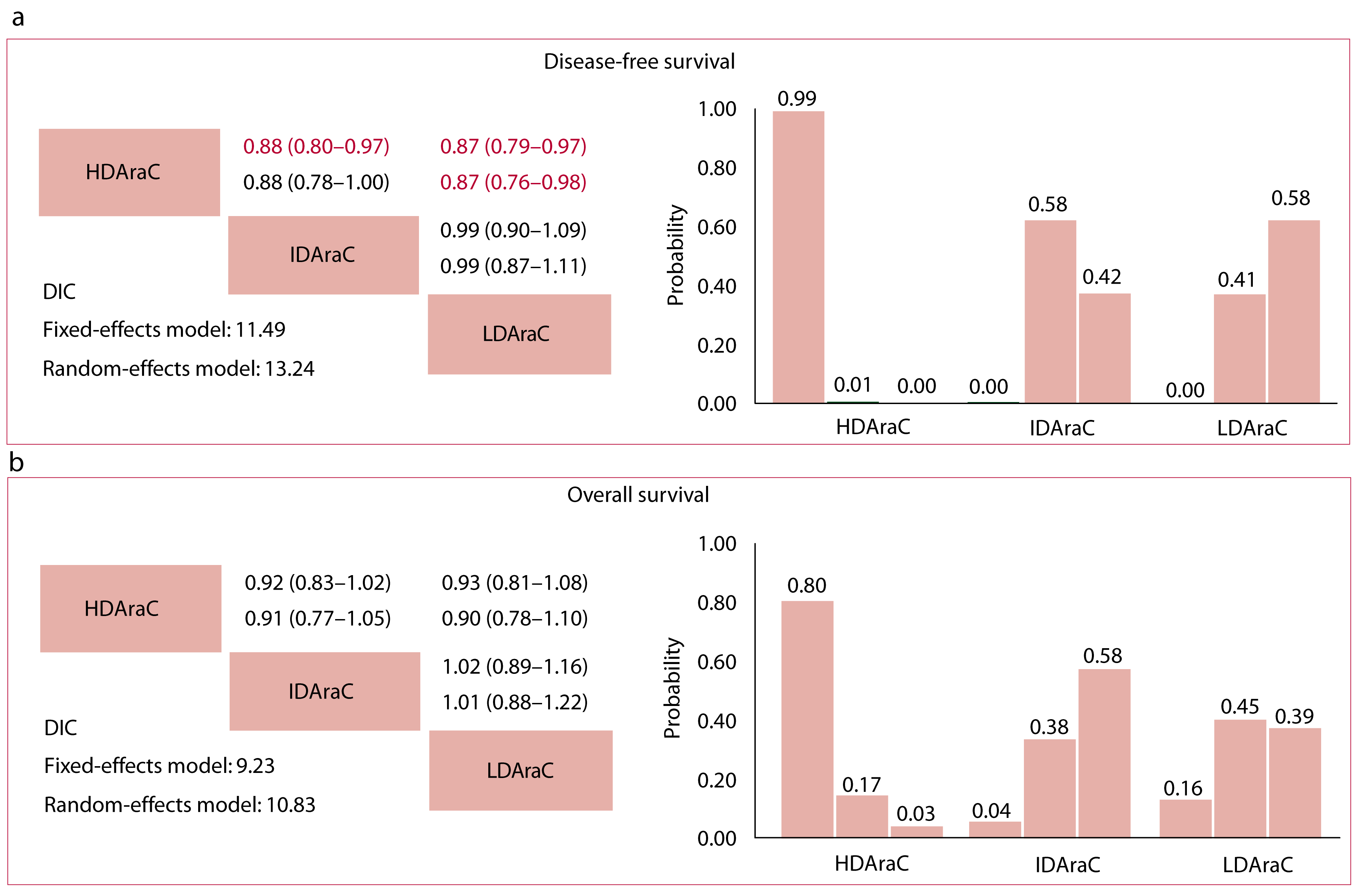
**Fig. S6**


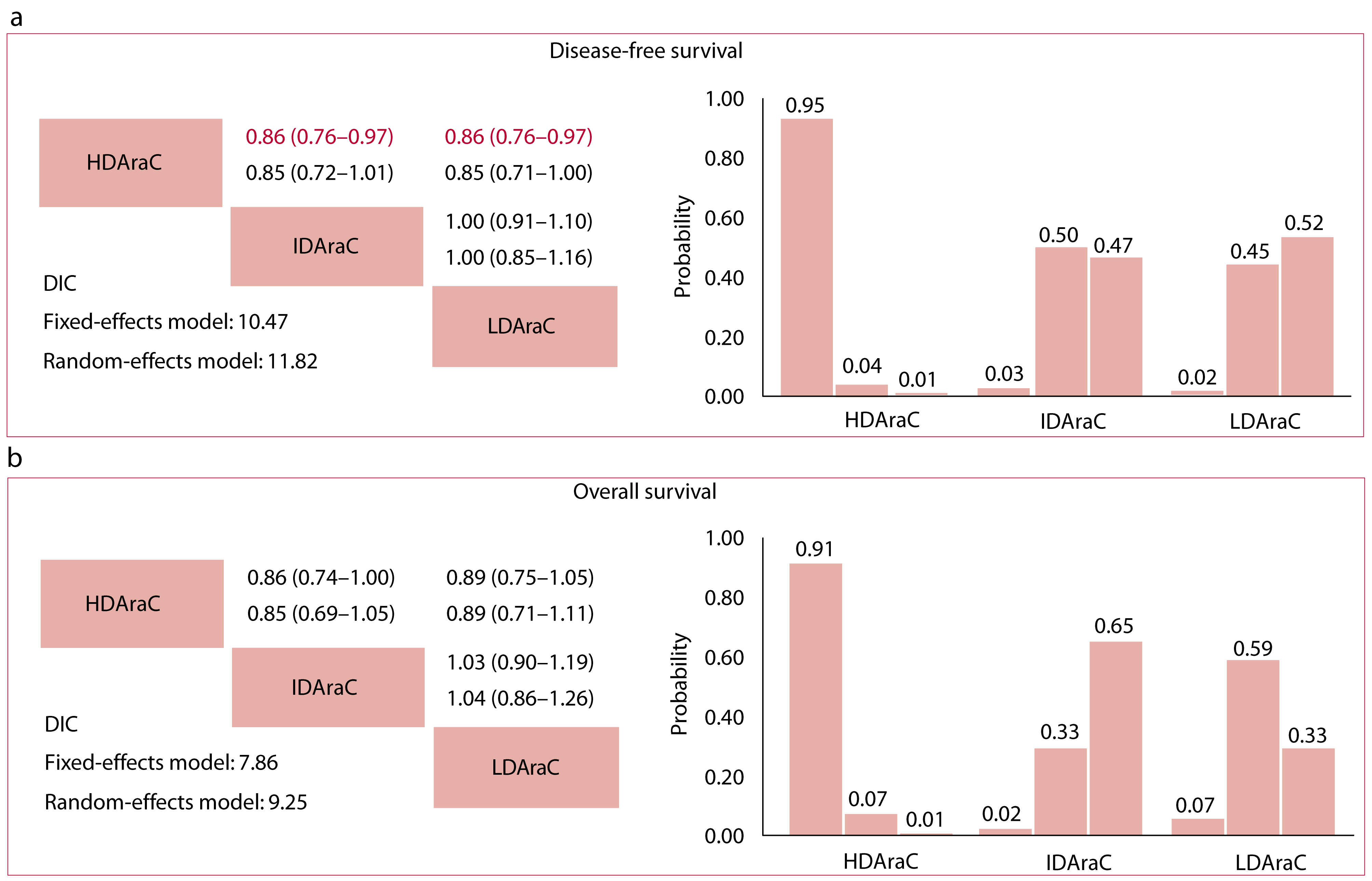
**Fig S7**

**Fig. S8**

**
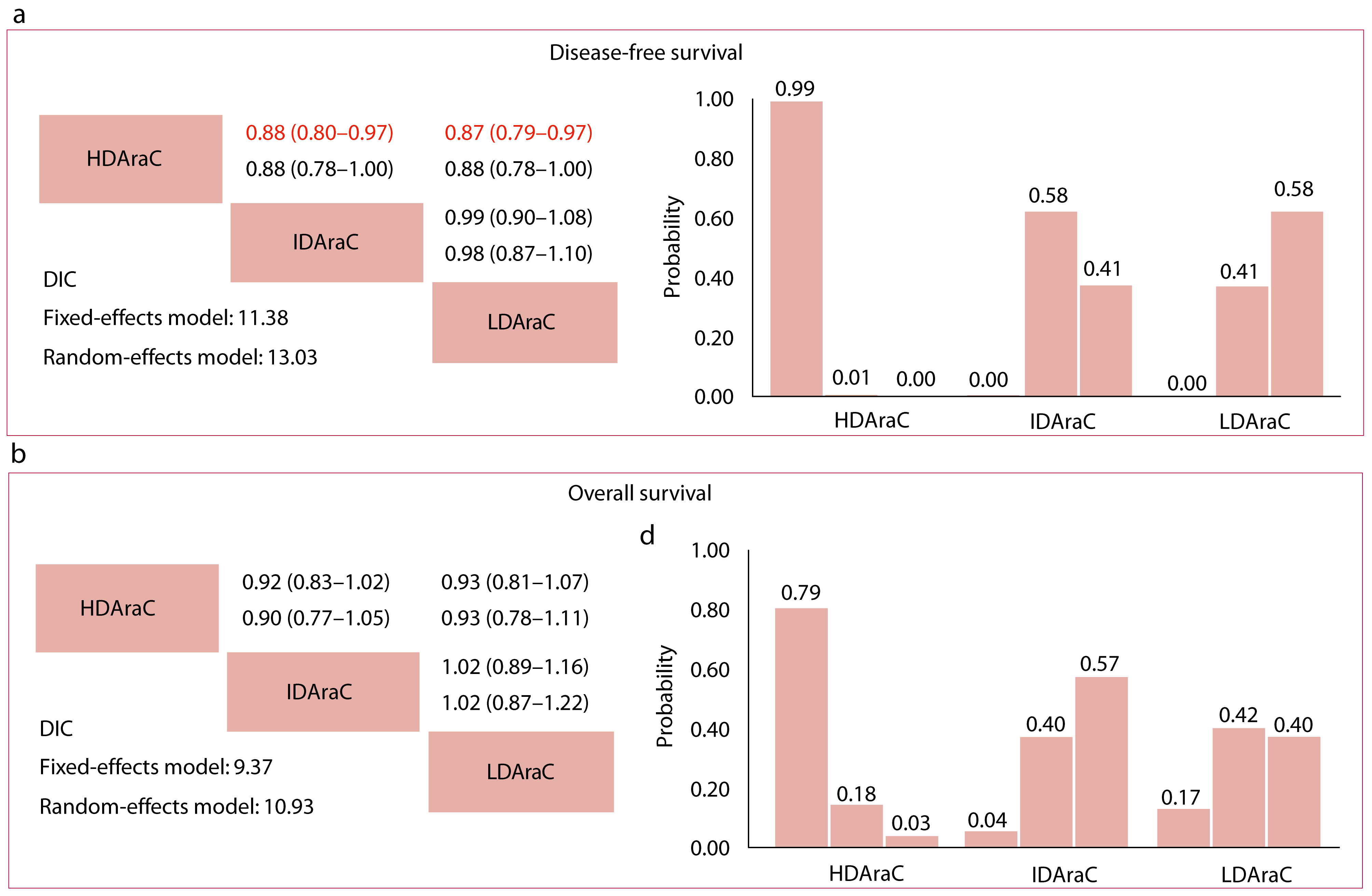
**


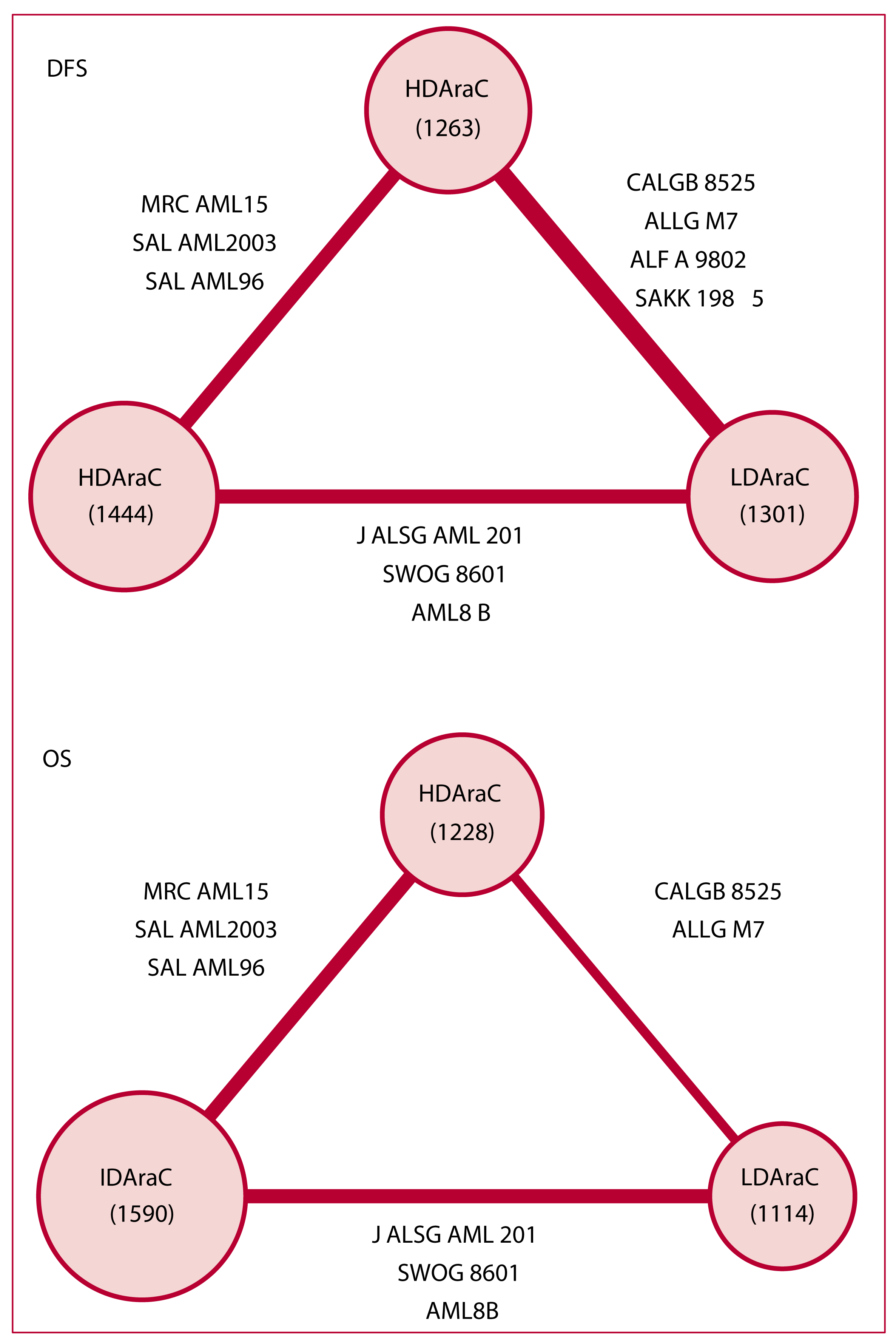
**Fig. S9**

**Table S1. Summary of studies included in the network meta-analysis (three trials that used non-conventional doses were excluded)**

| **Study** | **Design** | **Period** | **Entry Criteria** | **Size** | **Age**  **(years)** | **Induction**  **Therapy** | **CR**  **(%)** | **Single**  **Ara-C dose** |  | **Cumulative**  **Ara-C dose** | **Medium**  **follow**-**up**  **(Month)** |
| --- | --- | --- | --- | --- | --- | --- | --- | --- | --- | --- | --- |
| **HDAraC vs IDAraC** |  |  |  |  |  |  |  |  |  |  |  |
| MRC  AML15 | Open-label, multicenter phase III | 2002–2009 | Primary or secondary AML  including MDS;no pregnancy;  aged 15-60 years | 329  /328 | 48 (15–69) | DA or ADE  or FLAG-Ida × 1-2 | 78-  82 | (3 g/m^2^ every 12 h on days 1, 3, 5) × 2 vs (1.5 g/m^2^ every 12 h on days 1, 3, 5) × 2 |  | 36 g vs  18 g | 67 (2.4-114) |
| SAL  AML2003 | Open-label, multicenter phase III | 2003– 2009 | Primary or secondary AML, or refractory anemia with excess blasts (RAEB2); aged 16-60 years | 251  /254 | 47  (18–60) | DA × 2 | 65 | (3 g/m^2^ every 12 h on days 1, 3, 5) × 3 vs  (1 g/m^2^ every 12 h on days 1-5/6) × 2 |  | 54 g vs  20–22 g |  |
| **IDAraC**  **vs LDAraC** |  |  |  |  |  |  |  |  |  |  |  |
| SWOG  8601 | Open-label, multicenter phase III | 1986–  1993 | Primary AML; no MDS;  aged < 65 years; | 78  /53 | 45 (15–60) | DA | 58 | (2 g/m^2^ every 12 h on days 1-5) × 1 vs  (0.2 g/m^2^/d on days 1-7) × 2 |  | 20 g vs  2.8 g | 51  (NR) |
| JALSG  AML201 | Open-label, multicenter phase III | 2001–2005 | Primary AML with enough function  of major organs; no MDS;  aged 15-64 years | 389  /392 | 47 (15–64) | DA or AI  ×1-2 | 78 | (2 g/m^2^ every 12 h on days 1–5) × 3 vs  (0.2 g/m^2^/d on days 1-5) × 4 |  | 60 g vs  4 g | 48  (5-78) |
| EORTC &  GIMEMA  AML8B | Open-label, multicenter phase III | 1986–  1993 | Primary AML; no APL; absence of irreversible major organ failure;  aged 46-60 | 158  /157 | NR  (46–60) | DA ×1-2 | 61 | (0.5 g/m^2^ every 12 h on days 1-6) ×1 +  (2 g/m^2^ every 12 h on days 1-4) × 1 vs  (0.2 g/m^2^/d on days 1-7) × 1 |  | 22 g vs  1.4 g | 225  (NR) |
| **HDAraC vs LDAraC** |  |  |  |  |  |  |  |  |  |  |  |
| SAKK  1985 | Open-label, multicenter phase III | 1985–1992 | Primary AML (FAB Ml-6);  aged 15-65 years | 70  /67 | 45  (16–61) | DA ×1 +  (Amsacrine +  VP-16) ×1 | 61 | (3 g/m^2^ every 12 h on days 1-6) × 1 vs  (0.1 g/m^2^/d on days 1-7) × 1 |  | 36 g vs  0.7 g | 72  (NR) |
| CALGB  8525 | Open-label, multicenter phase III | 1985–1990 | Primary AML; no prior MDS,  uncontrolled infection;  aged 16-86 years | 187  /206  /203 | 52 (16–86) | DA × 1-2 | 64 | (3 g/m^2^ every 12 h on days 1, 3, 5) × 4 vs  (0.4 g/m^2^/d on days 1-5) × 4 |  | 72 g vs  8 g vs  2 g | 52 (NR) |

MRC, Medical Research Council; SAL, Study Alliance Leukemia; EORTC, European Organization for Research and Treatment of Cancer; GIMEMA, Gruppo Italiano Malattie Ematologiche Maligne dell’Adulto; JALSG, Japan Adult Leukemia Study; Group CALGB, Cancer and Leukemia Group B; AML, acute myeloid leukemia; MDS, myelodysplastic syndromes; Ara-C, cytarabine; DA, daunorubicin and cytarabine; ADE, cytarabine, daunorubicin, and etoposide; FLAG-Ida, fludarabine, cytarabine, granulocyte colony-stimulating factor, and idarubicin; MAV–MAMAC, mitoxantrone, standard-dose cytarabine, etoposide – intermediate-dose cytarabine, amsacrine; AI : cytarabine, idarubicin; IA, idarubicin; VP–16, etoposide; NR, not reported.

**Table S2.** Summary of studies included in the network meta-analysis (trials were divided by cumulative Ara-C dose)

| **Study** | **Design** | **Period** | **Entry Criteria** | **Size** | **Age**  **(years)** | **Induction**  **Therapy** | **CR**  **(%)** | **Singe**  **Ara-C dose** |  | **Cumulative**  **Ara-C dose** | **Medium**  **follow**-**up**  **(Month)** |
| --- | --- | --- | --- | --- | --- | --- | --- | --- | --- | --- | --- |
| **HDAraC vs IDAraC** |  |  |  |  |  |  |  |  |  |  |  |
| MRC  AML15 | Open-label, multicenter phase III | 2002–2009 | Primary or secondary AML  including MDS;no pregnancy;  aged 15-60 years | 329  /328 | 48 (15–69) | DA or ADE  or FLAG-Ida × 1-2 | 78-  82 | (3 g/m^2^ every 12 h on days 1, 3, 5) × 2 vs (1.5 g/m^2^ every 12 h on days 1, 3, 5) × 2 |  | 36 g vs  18 g | 67 (2.4-114) |
| SAL  AML2003 | Open-label, multicenter phase III | 2003– 2009 | Primary or secondary AML, or refractory anemia with excess blasts (RAEB2); aged 16-60 years | 251  /254 | 47  (18–60) | DA × 2 | 65 | (3 g/m^2^ every 12 h on days 1, 3, 5) × 3 vs  (1 g/m^2^ every 12 h on days 1-5/6) × 2 |  | 54 g vs  20–22 g |  |
| SAL  AML96 | Open-label, multicenter phase IV | 1996– 2003 | Primary or secondary AML; aged 15-64 years | 363  /382 | 47  (15–60) | MAV-MAMAC ×1 | 66 | (3 g/m^2^ every 12 h on days 1-6) × 1 vs  (1 g/m^2^ every 12 h on days 1-6) × 1 |  | 36 g vs  12 g | 99.6  (NR) |
| **IDAraC**  **vs LDAraC** |  |  |  |  |  |  |  |  |  |  |  |
| SWOG  8601 | Open-label, multicenter phase III | 1986–  1993 | Primary AML; no MDS;  aged < 65 years; | 78  /53 | 45 (15–60) | DA | 58 | (2 g/m^2^ every 12 h on days 1-5) × 1 vs  (0.2 g/m^2^/d on days 1-7) × 2 |  | 20 g vs  2.8 g | 51  (NR) |
| EORTC &  GIMEMA  AML8B | Open-label, multicenter phase III | 1986–  1993 | Primary AML; no APL; absence of irreversible major organ failure;  aged 46-60 | 158  /157 | NR  (46–60) | DA ×1-2 | 61 | ((0.5 g/m^2^ every 12 h on days 1-6) ×1 +  (2 g/m^2^ every 12 h on days 1-4) × 1 vs  (0.2 g/m^2^/d on days 1-7) × 1 |  | 22 g vs  1.4 g | 225  (NR) |
| **HDAraC vs LDAraC** |  |  |  |  |  |  |  |  |  |  |  |
| SAKK  1985 | Open-label, multicenter phase III | 1985–1992 | Primary AML (FAB Ml-6);  aged 15-65 years | 70  /67 | 45  (16–61) | DA ×1 +  (Amsacrine +  VP-16) ×1 | 61 | (3 g/m^2^ every 12 h on days 1-6) × 1 vs  (0.1 g/m^2^/d on days 1-7) × 1 |  | 36 g vs  0.7 g | 72  (NR) |
| CALGB  8525 | Open-label, multicenter phase III | 1985–1990 | Primary AML; no prior MDS,  uncontrolled infection;  aged 16-86 years | 187  /206  /203 | 52 (16–86) | DA × 1-2 | 64 | (3 g/m^2^ every 12 h on days 1, 3, 5) × 4 vs  (0.4 g/m^2^/d on days 1-5) × 4 |  | 72 g vs  8 g vs  2 g | 52 (NR) |
| ALFA  9802 | Open-label, multicenter phase III | 1999–2006 | Primary AML; no APL; absence of irreversible major organ failure;  aged 15-50 years | 117  /120 | 46 (17–50) | DA-MTZA  × 1 | 89 | (3 g/m^2^ every 12 h on days 1, 3, 5) × 4 vs  (0.5 mg/m^2^/d on days 1-3) × 2 |  | 72 g vs  3 g | 60  (NR) |
| ALLG  M7 | Open-label, multicenter phase III | 1995–2000 | Primary AML; absence of  irreversible major organ failure;  aged 15-60 years | 99  /103 | 41 (15–60) | High–dose  Ara-C + IA + VP-16 ×1-2 | 80 | (3 g/m^2^ every 12 h on days 1, 3, 5, 7) × 1 vs (0.1 g/m^2^/d on days 1-5) × 2 |  | 24 g vs  0.5 g | 45 (NR) |
| JALSG  AML201 | Open-label, multicenter phase III | 2001–2005 | Primary AML with enough function  of major organs; no MDS;  aged 15-64 years | 389  /392 | 47 (15–64) | DA or AI  ×1-2 | 78 | (2 g/m^2^ every 12 h on days 1-5) × 3 vs  (0.2 g/m^2^/d on days 1-5) × 4 |  | 60 g vs  4 g | 48  (5-78) |

MRC, Medical Research Council; SAL, Study Alliance Leukemia; EORTC, European Organization for Research and Treatment of Cancer; GIMEMA, Gruppo Italiano Malattie Ematologiche Maligne dell’Adulto; JALSG, Japan Adult Leukemia Study; Group CALGB, Cancer and Leukemia Group B; ALFA, Acute Leukemia French Association; ALLG, Australasian Leukaemia and Lymphoma Group; AML, acute myeloid leukemia; MDS, myelodysplastic syndromes; Ara-C, cytarabine; DA, daunorubicin and cytarabine; ADE, cytarabine, daunorubicin, and etoposide; FLAG-Ida, fludarabine, cytarabine, granulocyte colony-stimulating factor, and idarubicin; MAV–MAMAC, mitoxantrone, standard-dose cytarabine, etoposide – intermediate-dose cytarabine, amsacrine; AI : cytarabine, idarubicin; MTZA, mitoxantrone, cytarabine; IA, idarubicin; VP–16, etoposide; NR, not report.
